# AKT-dependent and -independent pathways mediate PTEN deletion-induced CNS axon regeneration

**DOI:** 10.1038/s41419-018-1289-z

**Published:** 2019-02-27

**Authors:** Haoliang Huang, Linqing Miao, Liu Yang, Feisi Liang, Qizhao Wang, Pei Zhuang, Yang Sun, Yang Hu

**Affiliations:** 1 Department of Ophthalmology, Stanford University School of Medicine, Palo Alto, CA 94304 USA; 2 Shriners Center for Neural Repair and Rehabilitation, Temple University School of Medicine, Philadelphia, PA 19140 USA

## Abstract

Phosphatase and tensin homolog (PTEN) acts as a brake for the phosphatidylinositol 3-kinase–AKT–mTOR complex 1 (mTORC1) pathway, the deletion of which promotes potent central nervous system (CNS) axon regeneration. Previously, we demonstrated that AKT activation is sufficient to promote CNS axon regeneration to a lesser extent than PTEN deletion. It is still questionable whether AKT is entirely responsible for the regenerative effect of PTEN deletion on CNS axons. Here, we show that blocking AKT or its downstream effectors, mTORC1 and GSK3β, significantly reduces PTEN deletion-induced mouse optic nerve regeneration, indicating the necessary role of AKT-dependent signaling. However, AKT is only marginally activated in PTEN-null mice due to mTORC1-mediated feedback inhibition. That combining PTEN deletion with AKT overexpression or GSK3β deletion achieves significantly more potent axonal regeneration suggests an AKT-independent pathway for axon regeneration. Elucidating the AKT-independent pathway is required to develop effective strategies for CNS axon regeneration.

## Introduction

Phosphatase and tensin homolog (PTEN) is a lipid phosphatase that acts as a brake for phosphatidylinositol 3-kinase (PI3K). Similar axon-regeneration phenotypes after PTEN deletion have been reported for mouse retinal ganglion cells (RGCs)^1^, cortical motor neurons^2^, drosophila sensory neurons^3^, and *Caenorhabditis elegans* motor neurons^4^, presumably through activating the PI3K pathway. The PI3K–AKT pathway is the central effector of multiple growth factors’ signaling to promote cell growth and survival^5^, mainly through the activation of mammalian target of rapamycin complex 1 (mTORC1), a master regulator of protein synthesis and cellular growth^6,7^. mTORC1-mediated activation of ribosomal protein S6 kinase 1 (S6K1) and inhibition of eukaryotic initiation factor 4E-binding proteins (4E-BPs) are critical for mRNA biogenesis, translation initiation, and elongation^8^.

AKT also phosphorylates and inhibits glycogen synthase kinase 3β (GSK3β), which is critical for neuronal polarization and axon branching, growth, and regeneration^9,10^. We have previously shown that S6K1 activation induces only very weak axon regeneration, whereas 4E-BP inhibition is necessary but not sufficient for axon regeneration^11^. Thus, in general, mTORC1 plays a necessary role in axon regeneration^12^. Interestingly, S6K1 also functions as a feedback inhibitor of PI3K signaling, which reduces AKT activation^13,14^. We found that a constitutive on S6K1 mutant inhibits AKT phosphorylation (pAKT) and decreases PTEN knockout (KO)-induced axon regeneration^11^, indicating the necessary role of AKT activation in axon regeneration. We further demonstrated that AKT activation induces central nervous system (CNS) axon regeneration through mTORC1 and GSK3β, two parallel and synergistical downstream effectors^10^.

It is still unknown whether AKT is the sole effector of PTEN KO for axon regeneration; it is only minimally activated in PTEN KO mice due to the feedback inhibition by mTORC1/S6K1. By exploiting RGCs and the crushed optic nerve (ON) as an in vivo axon injury model, we confirm the necessary role of AKT in PTEN deletion-induced axon regeneration, which acts through its downstream effectors mTORC1 and GSK3β. We also provide evidence that AKT-independent signaling is required to promote potent axon regeneration. Moreover, forced AKT activation or deletion of GSK3β further enhances PTEN deletion-induced axon regeneration, pointing toward more promising regeneration strategies based on targeting both AKT-dependent and -independent signals for CNS repair.

## Results

### AKT inhibition significantly reduces PTEN deletion-induced axon regeneration

**Fig. 1:**
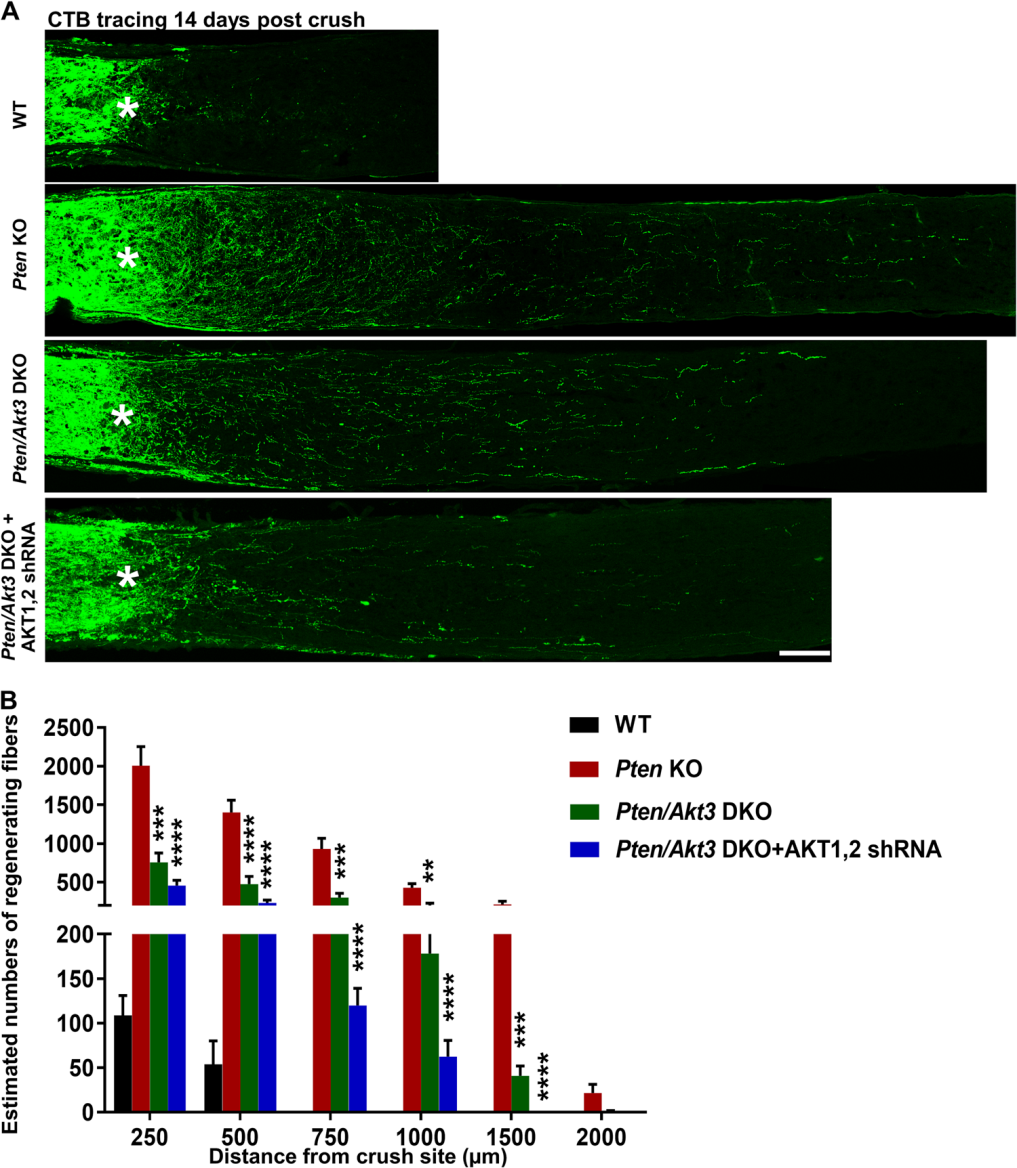
AKT inhibition significantly decreases the axon regeneration induced by PTEN deletion. **a** Confocal images of ON longitudinal sections showing regenerating fibers labeled with CTB-Alexa 488 2 weeks after ON crush. Scale bar, 100 µm. *Crush site. **b** Quantification of regenerating fibers at different distances distal to the lesion site. Data are presented as means ± s.e.m., *n* = 8–10. **p < 0.01, ***p < 0.001, ****p < 0.0001 versus Pten KO alone. One-way ANOVA with Bonferroni’s post hoc test. ANOVA, analysis of variance; CTB, cholera toxin β; ON, optic nerve

AKT3 is the major AKT isoform in RGCs and the most potent for axon regeneration^10^. To definitively determine the role of AKT in PTEN deletion-induced axon regeneration, we deleted AKT3 in PTEN KO mice. We crossed PTEN-floxed mice with AKT3 KO mice to generate PTEN/AKT3 double KO (DKO) RGCs after adeno-associated virus 2 (AAV2)-Cre intravitreal injection. We performed ON crush in these mice 2 weeks after AAV2-Cre injection. RGC axons that regenerated through the lesion site were labeled anterogradely by the intravitreal injection of the tracer Alexa 488-conjugated cholera toxin β (CTB). The axons were imaged and quantified in ON longitudinal sections at 14 days post crush (dpc): there was significantly less axon regeneration than in PTEN single KO mice (Fig. 1). Other AKT isoforms (AKT1 and AKT2) may have redundant roles on axon. Therefore, we generated AAV2 vectors containing a U6 promoter-driven shRNA construct that targeted a common sequence of AKT1 and AKT2 and a GFP construct for monitoring shRNA expression. We confirmed the total AKT KO effect by in situ hybridization with probes targeting AKT1-3 in AKT3 KO mice injected with AAV-U6-AKT1/2 shRNA (Supplementary Fig. 1). AAV2-U6-AKT1,2 shRNA intravitreal injection also further decreased, but did not totally abolish, axon regeneration in PTEN/AKT3 DKO mice (Fig. 1). Interestingly, AKT3 KO alone did not affect PTEN deletion-induced RGC survival, but blocking all the three isoforms of AKT significantly reduced RGC survival in PTEN KO mice (Supplementary Fig. 2A, B), indicating the redundant roles of AKT isoforms in RGC survival. However, some axons regenerated even with all the three AKT isoforms removed, suggesting an AKT-independent pathway. Thus, our data indicate that the three AKT isoforms act as redundant AKT signals that contribute to the effect of PTEN KO on axon regeneration and RGC survival.

### Marginal AKT activation after PTEN deletion in RGCs results in adequate mTORC1 activation for axon regeneration

**Fig. 2:**
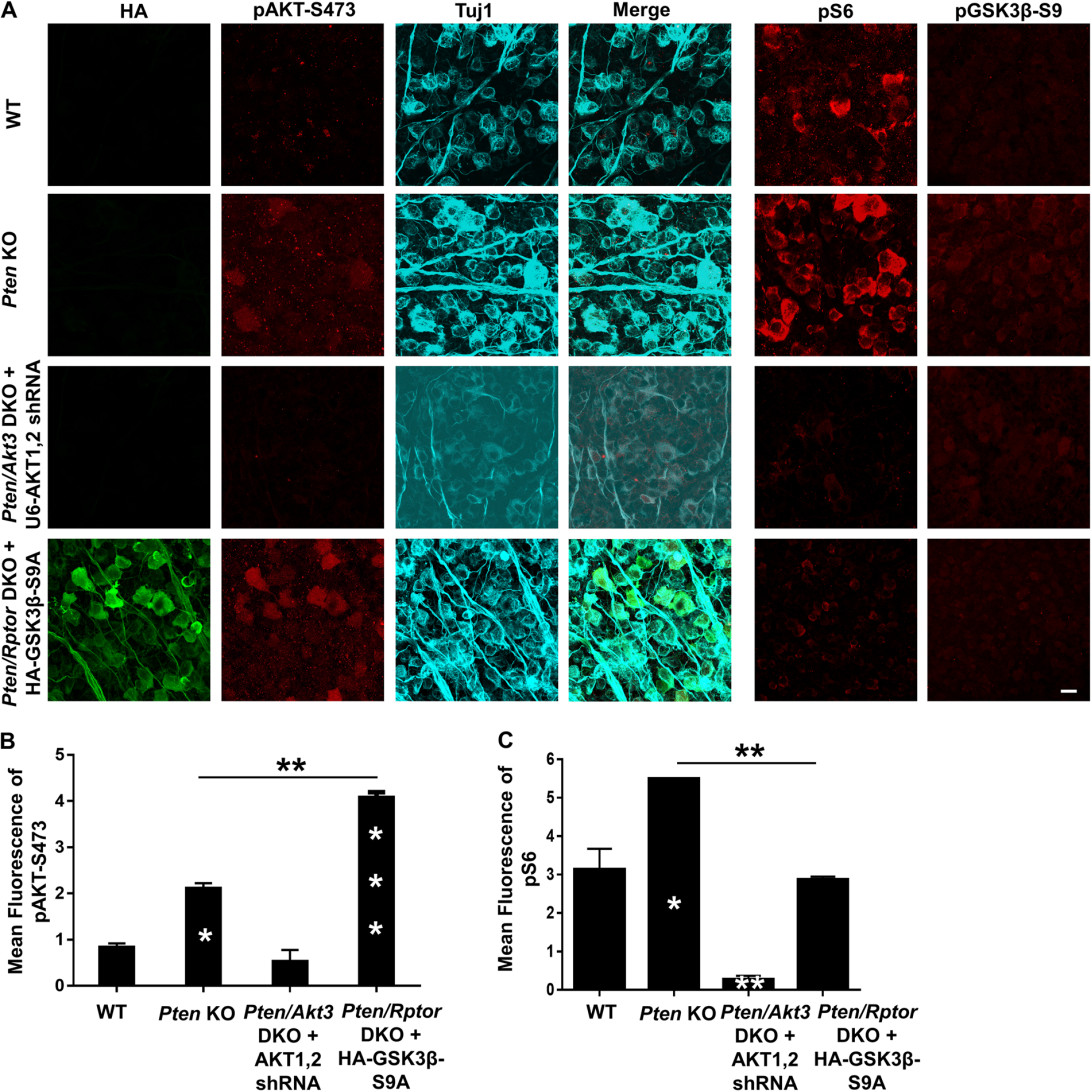
Activation of AKT and its downstream effectors mTORC1 and GSK3β in RGCs by PTEN deletion and/or mTORC1 inhibition. **a** Confocal images of flat-mounted retinas showing co-labeling of HA tag, Tuj1 as a marker of RGCs, pAKT-S473, and their merged images, and phosphorylation of S6 and GSK3β-S9 in a separate retina sample. Scale bar, 20 µm. **b** Mean fluorescence intensities of pAKT-S473 and **c** mean fluorescence intensities of pS6. Data are presented as means ± s.e.m, *n* = 3. *p < 0.05, **p < 0.01, ***p < 0.001; white stars versus WT mice. One-way ANOVA with Tukey’s multiple comparison post hoc test. ANOVA, analysis of variance; RGCs, retinal ganglion cells

**Fig. 3:**
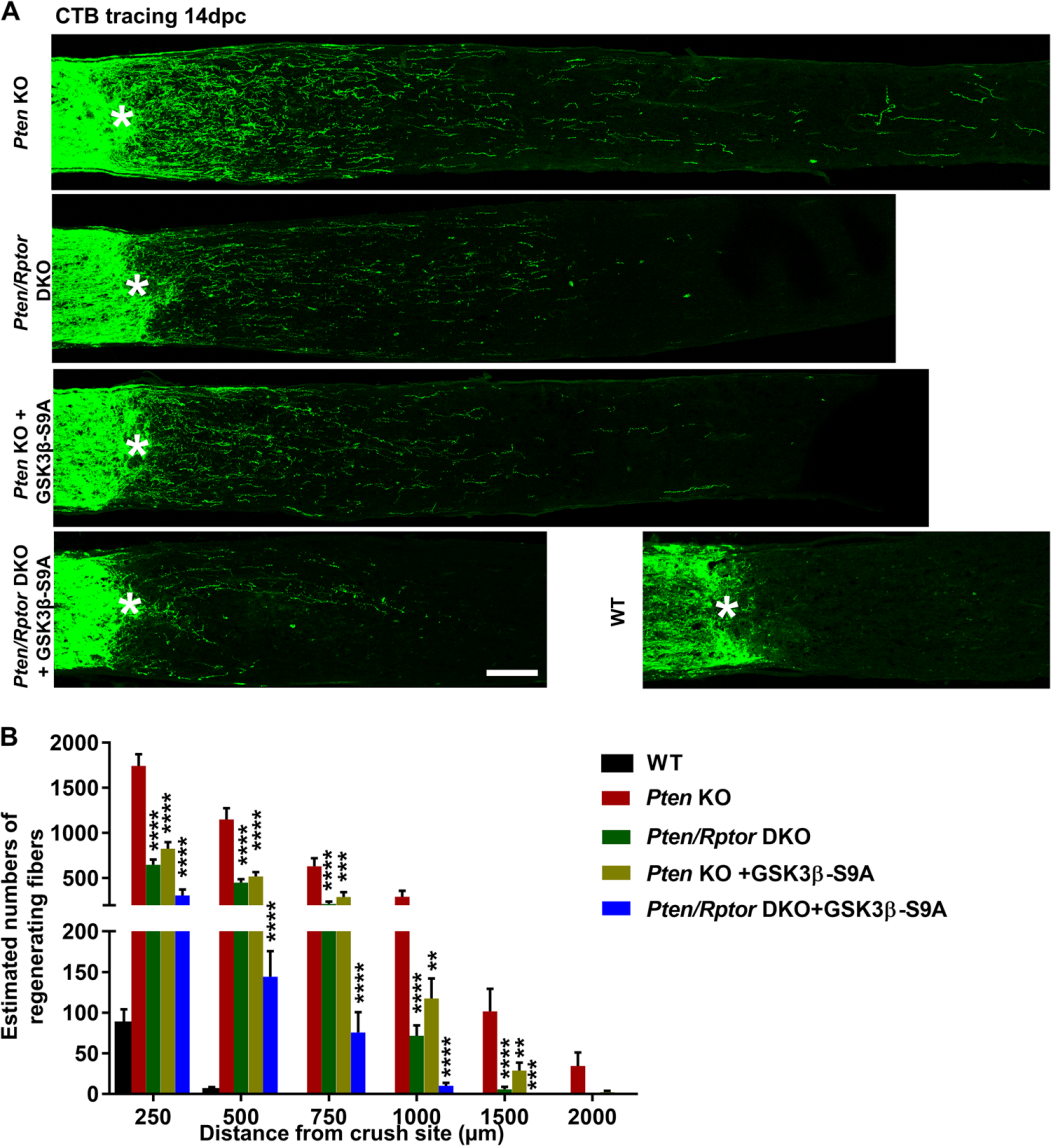
Blocking mTORC1 and/or GSK3β-S9 phosphorylation significantly inhibits PTEN KO-induced axon regeneration. **a** Confocal images of ON longitudinal sections showing regenerating fibers labeled with CTB-Alexa 488 2 weeks after ON crush. Scale bar, 100 µm. *Crush site. **b** Quantification of regenerating fibers at different distances distal to the lesion site. Data are presented as means ± s.e.m., *n* = 11–20. **p < 0.01, ***p < 0.001, ****p < 0.0001 versus Pten KO alone. One-way ANOVA with Bonferroni’s post hoc test. ANOVA, analysis of variance; CTB, cholera toxin β; ON, optic nerve

Next, we examined AKT activation in whole mounts of PTEN KO RGCs. pAKT was only slightly increased, but mTORC1 was activated robustly, indicated by the significantly increased phosphorylation of ribosome protein S6 (pS6), which is a substrate of S6K1 and has often served as a marker for mTORC1 activation (Fig. 2). The low AKT activation was responsible for mTORC1 activation because blocking AKT by AKT3 deletion and AKT1/2 shRNA significantly decreased both pAKT and pS6 in PTEN KO mice (Fig. 2). To test whether mTORC1 limits AKT activation through feedback inhibition^7,11^, we targeted *Rptor* (regulatory associated protein of mTOR), which is unique to mTORC1 and the deletion of which blocks mTORC1 activity^7,15^. Destruction of mTORC1 by AAV2-Cre-mediated deletion of *Rptor* and *Pten* in *Rptor/Pten* double-floxed mice significantly decreased pS6 but increased pAKT (Fig. 2), indicating a feedback loop in PTEN KO RGCs. Thus, the balance between PTEN KO forward activation and mTORC1 feedback inhibition results in limited AKT activation but reasonable mTORC1 activation, which may be essential for axon regeneration. To confirm this notion, we performed an ON crush injury in *Pten/Rptor* DKO mice and, as expected, found that *Rptor* deletion (mTORC1 inhibition) significantly decreased PTEN KO-induced axon regeneration (Fig. 3). This result is consistent with our previous finding that mTORC1 is necessary for axon regeneration^11,12^. Taken together, these observations led us to conclude that PTEN deletion results in slight AKT activation, which activates mTORC1 adequately to allow axon regeneration.

### AKT-induced GSK3β phosphorylation/inhibition is also necessary for PTEN deletion-induced axon regeneration

We have shown before that mTORC1 activation and GSK3β S9 phosphorylation/inhibition contribute synergistically to AKT-induced axon regeneration^10^. To further confirm the role of GSK3β S9 phosphorylation/inhibition in PTEN KO-induced axon regeneration, we prepared an AAV2-GSK3β-S9A mutant that cannot be phosphorylated by AKT and therefore cannot be inhibited after PTEN deletion. We then performed an ON crush injury in PTEN KO mice with GSK3β-S9A overexpression (constitutive on GSK3β) and found significantly decreased axon regeneration (Fig. 3). In addition, combining *Rptor* deletion and GSK3β-S9A overexpression further decreased, but did not totally abolish, PTEN KO-induced axon regeneration (Fig. 3), indicating the synergistic effects of these two parallel downstream effectors of AKT and also suggesting the possibility of AKT-independent signaling in PTEN deletion-induced axon regeneration. Unfortunately, because the phosphor-GSK3β-S9 antibody does not produce dependable immunostaining when the pGSK3β-S9 level is low, we detected hardly any increase of pGSK3β-S9 in *Pten* KO RGCs compared to wild-type (WT) RGCs (Fig. 2) and were unable to detect a further decrease with GSK3β-S9A overexpression. This result suggests, however, that the low activation of AKT in PTEN KO mice induces only a low level of pGSK3β-S9 and that this amount is necessary for axon regeneration. Surprisingly, neither mTORC1 inhibition nor GSK3β-S9A overexpression affects PTEN KO-induced RGC protection (Supplementary Fig. 2C, D), implying that other effectors downstream of AKT contribute to RGC survival. These results are additional evidence for the partial but essential role of AKT and its downstream effectors (mTORC1 and GSK3β) in PTEN KO-induced axon regeneration.

### Robust AKT activation or GSK3β deletion promotes more potent axon regeneration in Pten KO mice

**Fig. 4:**
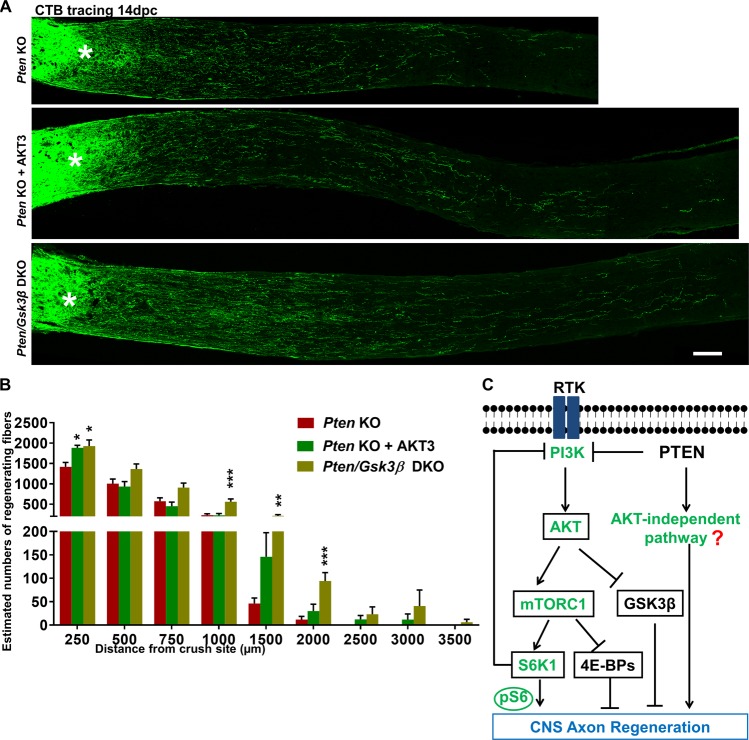
AKT3 overexpression or GSK3β deletion further increases PTEN KO-induced axon regeneration. **a** Confocal images of ON longitudinal sections showing regenerating fibers labeled with CTB-Alexa 488 2 weeks after ON crush. Scale bar, 100 µm. *Crush site. **b** Quantification of regenerating fibers at different distances distal to the lesion site. Data are presented as means ± s.e.m., *n* = 11–13. *p < 0.05, **p < 0.01, ***p < 0.001 versus Pten KO alone. One-way ANOVA with Bonferroni’s post hoc test. **c** A schematic illustration of AKT-dependent and -independent pathways downstream of PTEN deletion that act additively to promote CNS axon regeneration. PTEN deletion minimally activates AKT due to the feedback inhibition of mTORC1/S6K1. The low AKT activation results in adequate mTORC1 activation and low GSK3β inhibition, which are necessary for axon regeneration. The question mark represents unknown effectors of PTEN deletion, which are AKT independent and cooperate with AKT-dependent signaling to promote axon regeneration. ANOVA, analysis of variance; CTB, cholera toxin β; ON, optic nerve

Since AKT is only marginally activated in PTEN KO mice (Fig. 2) and we have previously shown that sustained potent activation of AKT3 in RGCs by AAV2-myr-AKT3 injection promotes significant axon regeneration^10^, we used the same AAV vectors to overcome the feedback inhibition of mTORC1 and, therefore, to force AKT activation in PTEN KO mice. As expected, AAV2-myr-AKT3 intravitreal injection significantly increased the phosphorylation levels of AKT and GSK3β S9 (Supplementary Fig. 3) (please note that only overexpression of AKT can achieve this reliable pGSK3β S9 labeling) and resulted in significantly more and longer regenerating axons than PTEN KO alone (Fig. 4a, b). Similarly, AAV2-Cre-mediated *Pten/Gsk3β* DKO also resulted in more potent axon regeneration than PTEN KO alone (Fig. 4a, b). Fully activated AKT or the total deletion of GSK3β, therefore, acts synergistically with AKT-independent signaling downstream of PTEN deletion to promote CNS axon regeneration.

## Discussion

The results of our present and earlier studies using genetic manipulations in RGCs specifically provide a molecular dissection of the PTEN–PI3K–AKT–mTORC1–GSK3β signaling pathway and definitively determine the linear and parallel signals that contribute to CNS axon regeneration (Fig. 4c). We have previously demonstrated that mTORC1 and its downstream effectors, S6K1 and 4E-BP, are necessary for PTEN KO-induced axon regeneration^11,12^. This conclusion led us to propose that a key proactive signal originating downstream from PTEN triggers the neuron intrinsic-growth machinery^12^. We later showed that AKT activation alone is sufficient to promote axon regeneration through the modulation of two parallel pathways, mTORC1 and GSK3β, and that the regeneration is less extensive than that of PTEN deletion^10^. Thus, AKT is a downstream effector of PTEN but cannot fully account for the effect of PTEN deletion on axon regeneration. The results of the present study confirm that AKT is only marginally activated after PTEN deletion due to mTORC1/S6K1-mediated feedback inhibition (Fig. 2), which is consistent with our previous observation that constitutive S6K1 activation decreases pAKT and axon regeneration in PTEN KO mice^11^. A similar effect has also been found in *C. elegans*^16^, and the inhibition of S6K1 has recently been shown to promote regeneration in mouse spinal cord^17^. We further demonstrated that the low AKT activation results in adequate, but not robust, mTORC1 activation and GSK3β inhibition, which are necessary for PTEN deletion-induced CNS axon regeneration. We also confirmed the functional redundancy of AKT isoforms in mediating PTEN effect, as in other systems^18^.

Clearly, however, these low levels of AKT activation and GSK3β inhibition are insufficient for axon regeneration. PTEN deletion must function through an AKT-independent pathway, which stimulates axon regeneration, but needs to cooperate with AKT-dependent signals to release the full potential of PTEN deletion. This uncharacterized pathway acts in parallel or by cross talk with the AKT-dependent pathway because potent AKT activation or total GSK3β inhibition further enhances regeneration in PTEN KO mice (Fig. 4c). Interestingly, AKT-independent GSK3β inhibition has been shown in peripheral axon regeneration^19^, and it will be interesting to determine whether the downstream effectors of GSK3β signaling comprise the nodal point that connects with PTEN deletion-induced AKT-independent signaling. Because the major cellular function of PTEN is to dephosphorylate PIP3 to PIP2^5^, PTEN deletion will increase PIP3 and, therefore, activate PIP3-dependent signaling, including many AKT-independent pathways^20^. In addition, PTEN can dephosphorylate focal adhesion kinase (FAK) and Shc, and the deletion of PTEN activates FAK, RAS, and ERK^21^. Furthermore, PTEN is also present in the nucleus, where it is proposed to play a non-catalytic role in chromosomal instability and DNA repair^22,23^. Elucidation of these PTEN-dependent but AKT-independent pathways and the mechanisms by which they are cross regulated with the AKT-dependent pathway is likely to lead eventually to safe and effective regenerative strategies for CNS repair.

## Methods and materials

### Mice

*Pten*^flox/flox^ and *Rptor*^flox/flox^ mice with C57BL/6 background and C57BL/6 WT mice were purchased from Jackson Laboratories (Bar Harbor, Maine). *Gsk3b*^flox/flox^ mice with C57BL/6 background were originally developed by Dr. Jim Woodgett^24,25^ and were acquired from Dr. Thomas Force. AKT3 KO mice were acquired from the Dr. Morris Birnbaum lab^26^. Either sex was randomly used in experiments. All experimental procedures were performed in compliance with the animal protocols approved by the IACUC at Stanford University School of Medicine. For all surgical and treatment comparisons, control and treatment groups were prepared together in single cohorts, and the experiment was repeated at least twice.

### Constructs

AAV-Myr-3HA-AKTs, AAV-3HA-GSK3β-S9A, and AAV-Cre have been described before^10^.

### AAV production

The detailed procedure has been described previously^11,27^. Briefly, AAV plasmids containing the transgenes were co-transfected with pAAV2 (pACG2)-RC triple mutant (Y444, 500, 730F)^28–30^ and the pHelper plasmid (Stratagene) into HEK293T cells. Seveny-two hours after transfection, the cells were lysed to release the viral particles, which were precipitated by 40% polyethylene glycol and purified by cesium chloride density-gradient centrifugation. The fractions with refractive index from 1.370 to 1.374 were taken out for dialysis in a MWCO 7000 Slide-A-LYZER cassette (Pierce) overnight at 4 °C. The AAV titers that we used for this study were in the range of 1.5–2.5 × 10^12^ genome copies/ml, determined by real-time PCR.

### Intravitreal injection and ON crush

Mice were anesthetized by xylazine and ketamine based on their body weight (0.01 mg xylazine/g + 0.08 mg ketamine/g). For each AAV intravitreal injection, a micropipette was inserted into the peripheral retina of 3-week-old mice just behind the ora serrata and advanced into the vitreous chamber so as to avoid damage to the lens. Approximately 2 µl of the vitreous was removed before injection of 2 µl AAV into the vitreous chamber. Roughly 3 × 10^9^ vector genome/retina routinely achieves more than 80% RGC transduction, as assessed by Cre-dependent reporter mouse line^11^. ON crush was performed 2 weeks after AAV injection (5weeks old): the ON was exposed intraorbitally and crushed with a jeweler’s forceps (Dumont #5; Fine Science Tools, Foster City, CA, USA) for 5 s approximately 0.5 mm behind the eyeball. Care was taken not to damage the underlying ophthalmic artery. Eye ointment containing neomycin (Akorn, Somerset, NJ, USA) was applied to protect the cornea after surgery.

### RGC axon anterograde tracing

Two microliters of CTB conjugated with fluorescence Alexa-488 (2 μg/μl; Invitrogen) was injected into the vitreous chamber 2 days before euthanizing the animals at 7 weeks old (2 weeks after ON crush) to label the regenerating axons in the ON. Animals were euthanized by CO_2_ and fixed by perfusion with 4% paraformaldehyde in cold phosphate-buffered saline (PBS). Eyes with the nerve segment still attached were dissected out and post-fixed in the same fixative for another 2 h at room temperature. Tissues were cryoprotected with increasing concentrations of sucrose (15–30%) and optimal cutting temperature compound (Tissue Tek). They were then snap frozen in dry ice, and serial longitudinal cross sections (8 µm) were cut and stored at −80 °C until processed.

### Immunohistochemistry of flat-mount retina

Retinas were dissected out from 4% paraformaldehyde (PFA)-fixed eyes and washed extensively in PBS before blocking in staining buffer (10% normal goat serum and 2% Triton X-100 in PBS) for 30 min. Mouse or rabbit antibodies for neuronal class ß-III tubulin (clone Tuj1, 1:500; Covance), rat HA (clone 3F10, 1:200; Roche), phospho-S6-Ser240/244 (1:200, #5364; Cell Signaling), phospho-AKT-Ser473 (1:200, #4058; Cell Signaling), pan AKT (1:200, #2920; Cell Signaling), and phospho-GSK-3β (Ser9) (1:100, #9323; Cell Signaling) were diluted in the same staining buffer. RBPMS guinea pig antibody was made at ProSci, CA, according to publications^31,32^. Floating retinas were incubated with primary antibodies overnight at 4 °C and washed three times for 30 min each with PBS. Secondary antibodies (Cy2, Cy3, or Cy5 conjugated) were then applied (1:200; Jackson ImmunoResearch) and incubated for 1 h at room temperature. Retinas were again washed three times for 30 min each with PBS before a cover slip was attached with Fluoromount-G (SouthernBiotech).

### Counting surviving RGCs and regenerating axons

For RGC counting, whole-mount retinas were immunostained with the Tuj1 antibody, and 6–9 fields were randomly sampled from the peripheral regions of each retina. All the Tuj1^**+**^ RGCs were counted from each field to generate the average surviving RGC number/retina. The percentage of RGC survival was calculated as the ratio of surviving RGC numbers in injured eyes to those in contralateral uninjured eyes. For axon counting, the number of CTB-labeled axons was quantified as described previously^1,11,33^. Briefly, we counted the fibers that crossed the perpendicular lines drawn on the ON sections distal to the crush site in increments of 250 µm till 1000 µm, and then every 500 µm till no fibers were visible. The width of the nerve (*R*) was measured at the point (d), at which the counts were taken, and used together with the thickness of the section (*t* = 8μm) to calculate the number of axons per µm^2^ area of the nerve. The formula used to calculate is ∑*a*_d_ = *πr*^2^× (axon number)/(*R* × *t*). The total number of axons per section was then averaged over threesections per animal. All CTB signals that were in the range of intensity that was set from the lowest intensity to the maximum intensity after background subtraction were counted as individual fibers by Nikon NIS Element R4 software. The investigators who counted the cells or axons were blinded to the treatment of the samples.

### Fluorescent in situ hybridization with retina cross sections

Adult mice were perfused with ice-cold 4% PFA/PBS, and the eyeballs were dissected out and fixed in 4% PFA/PBS at 4 °C overnight. The eyeballs were dehydrated with increasing concentrations of sucrose solution (15–30%) overnight before embedding in optimal cutting temperature compound on dry ice. Serial cross sections (12 µm) were cut with a Leica cryostat and collected on Superfrost Plus Slides. The sections were pretreated with protease and then subjected to in situ hybridization with RNAscope Multiplex Fluorescent Detection Reagents V2 according to the manufacturer’s instruction (Advanced Cell Diagnostics, Hayward, CA, USA). Briefly, the sections were hybridized with the probe solution, followed by amplification and probe detection using TSA plus fluorescein/cyanine 5 (PerkinElmer, San Jose, CA, USA). The sections were mounted with Fluoromount-G (SouthernBiotech, Birmingham, AL, USA). Images were captured by Zeiss LSM 880 confocal laser scanning microscope with 63 × /1.40 Oil DIC (Carl Zeiss Microscopy, Thornwood, NY, USA). RNAscope probes Mm-Akt1, Mm-Akt2, and Mm-Akt1 were purchased from ACD and targeted bases 1130–2560, 2618–3665, and 24–1138 of mouse Akt1, Akt2, and Akt3 mRNA (NCBI reference sequence: NM_009652.3, NM_007434.4, and NM_011785.3), respectively.

### Statistical analyses

GraphPad Prism 6 was used to generate graphs and for statistical analyses. Data are presented as means ± s.e.m. One-way analysis of variance with post hoc test was used for multiple comparisons.

## Supplementary information

AKT-Dependent and Independent Pathways Mediate PTEN Deletion-Induced CNS Axon Regeneration
