# Supplementary material for: AKT-dependent and -independent pathways mediate PTEN deletion-induced CNS axon regeneration
Source: Cell Death Dis. 2019 Feb 27;10(3):203. doi: 10.1038/s41419-018-1289-z (PMC6393504; doi:10.1038/s41419-018-1289-z)
Supplement: Supplementary file 1 — AKT-Dependent and Independent Pathways Mediate PTEN Deletion-Induced CNS Axon Regeneration [file 41419_2018_1289_MOESM1_ESM.docx]

AKT-Dependent and Independent Pathways Mediate PTEN Deletion-Induced CNS Axon Regeneration

**SUPPLEMENTARY FIGUREs and LEGENDS**


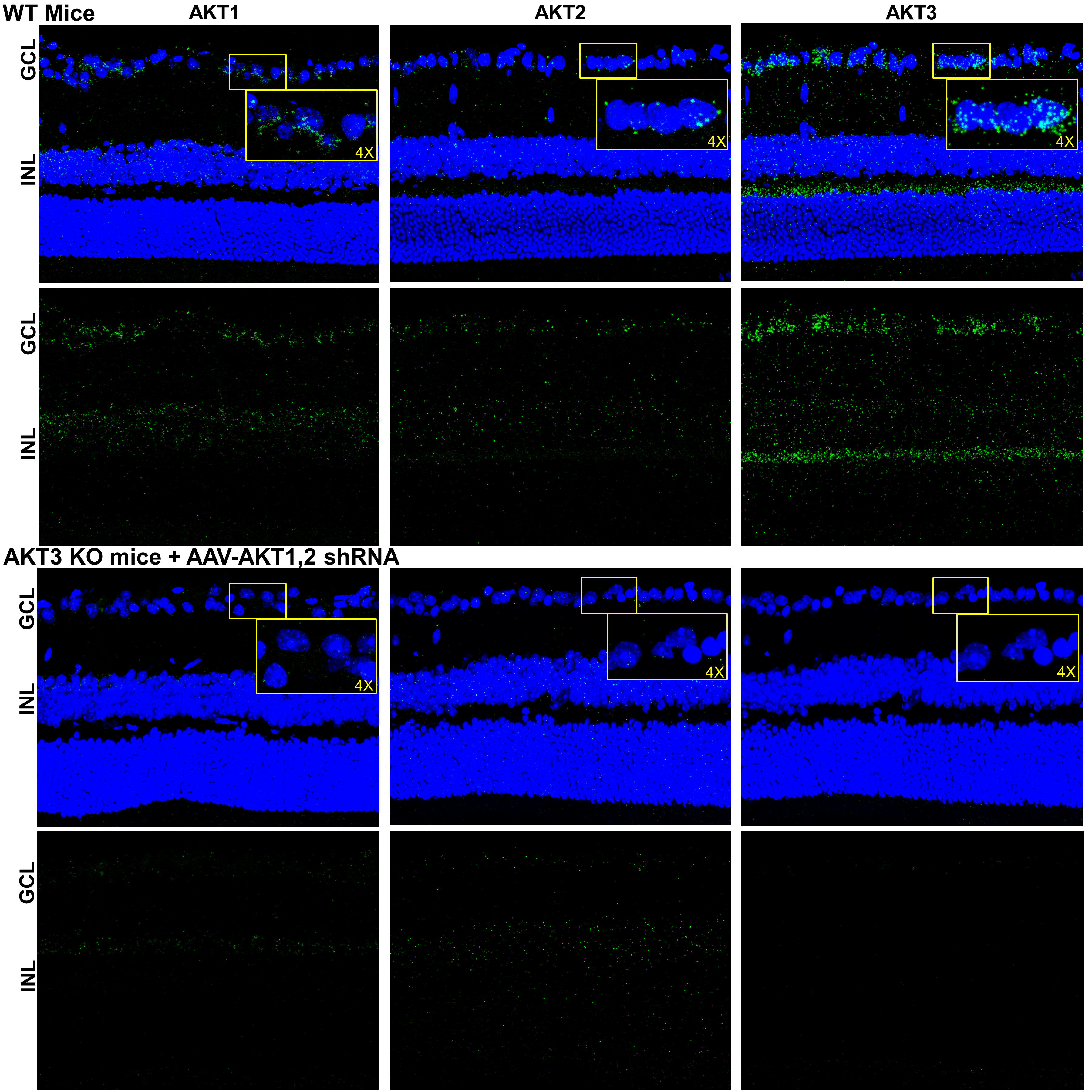


**Figure S1. The KO effect of AKT1-3 by AAV-AKT1/2 shRNA in AKT3 KO mice.** Confocal images of retina cross sections showing Dapi-labeled nuclei (blue) and AKTs’ mRNAs detected by fluorescent in situ hybridization (pseudocolored with green).


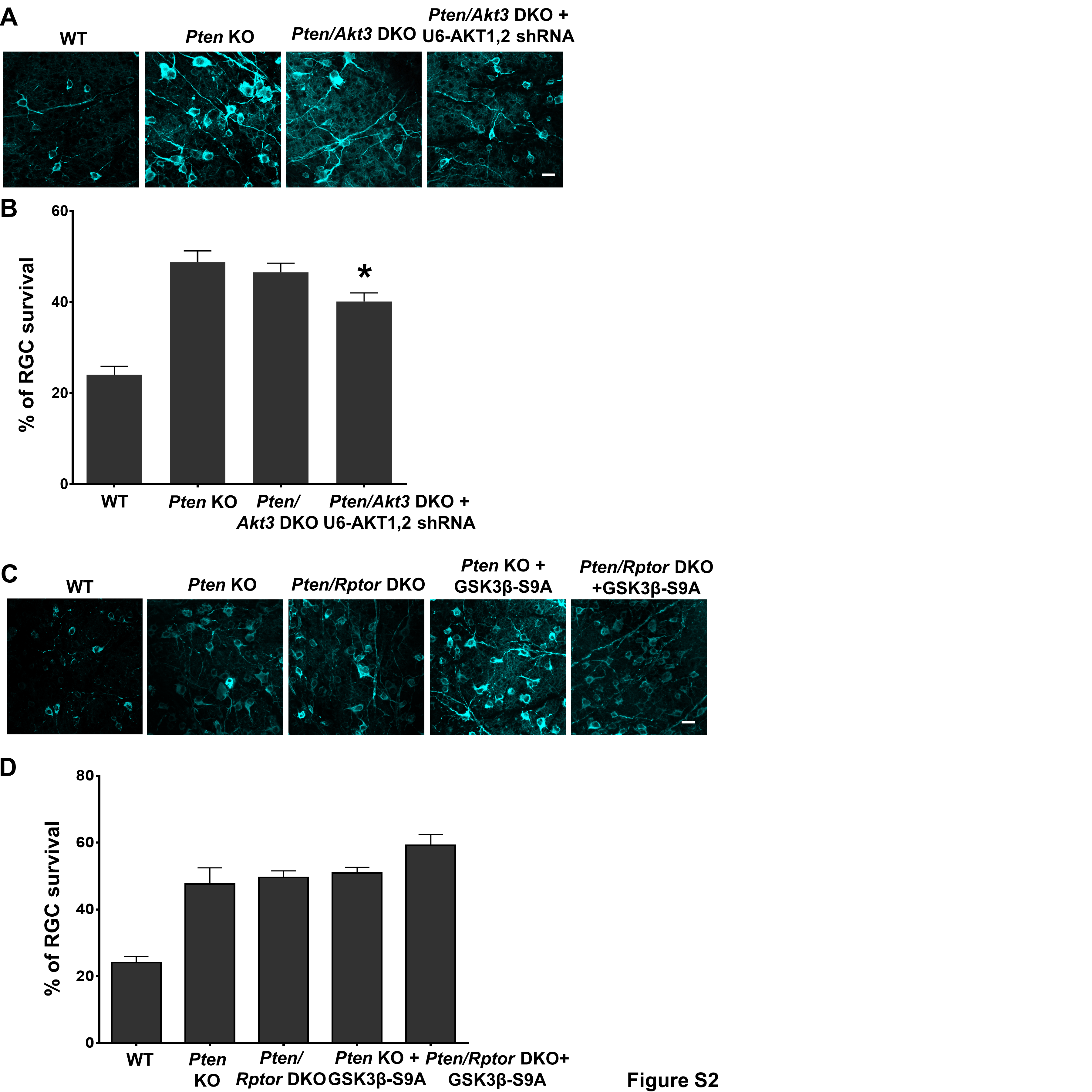


**Figure S2. The effects of AKT, Rptor and GSK3β manipulation on PTEN KO-induced RGC survival. A, C,** Confocal images of flat-mounted retinas showing Tuj1 positive RGCs, 2 weeks after ON crush. Scale bar, 20 µm. **B, D,** Quantification of surviving RGCs, represented as percentage of Tuj1 positive RGCs in the injured eye, compared to the intact contralateral eye. Data are presented as means ± s.e.m, n=9. *: p<0.05 versus Pten KO alone. One-way ANOVA with Dunnett’s post hoc test.


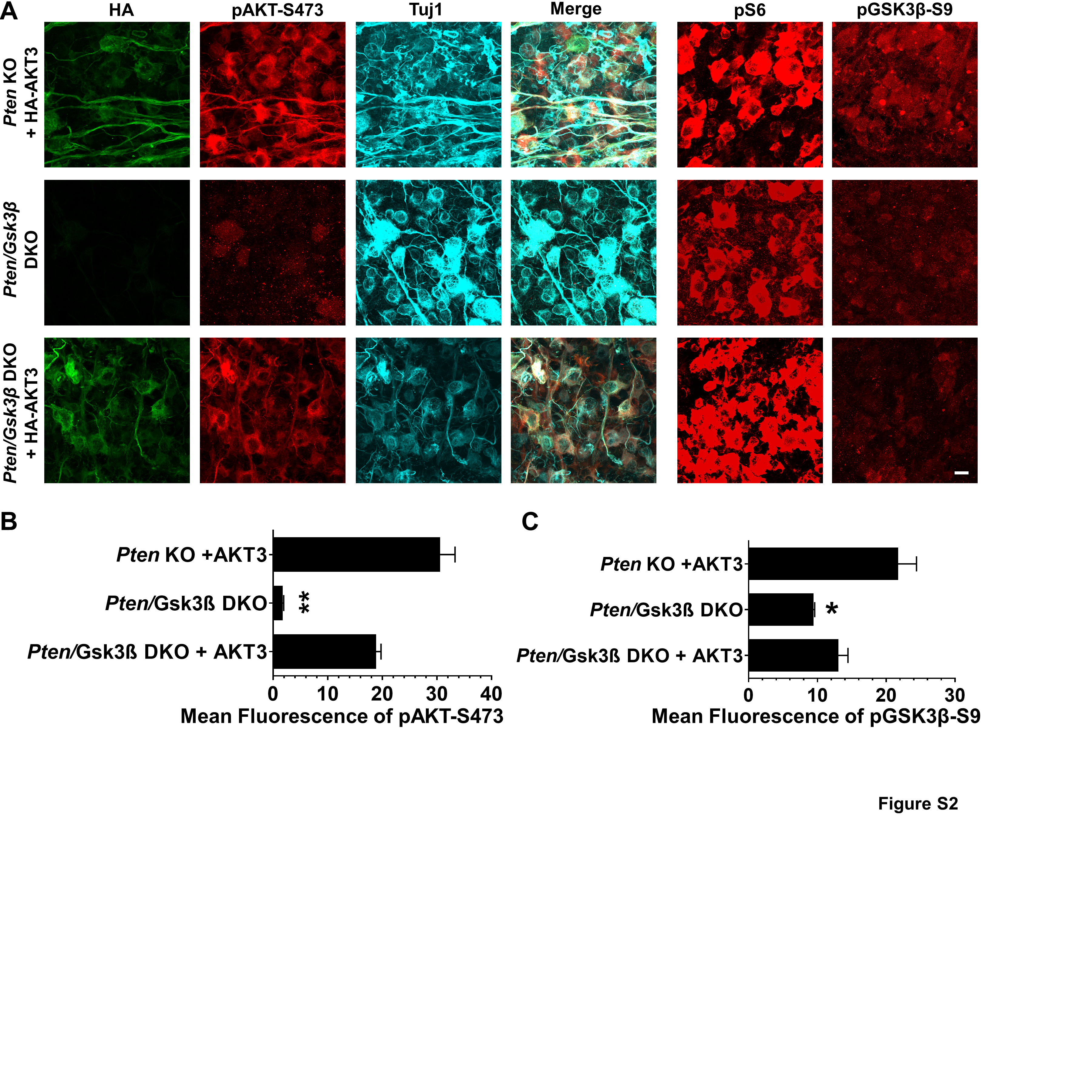


**Figure S3. Activation of AKT and its downstream effectors mTORC1 and GSK3β in RGCs by PTEN deletion, AKT3 overexpression and GSK3β deletion. A,** Confocal images of flat-mounted retinas showing co-labeling of HA tag, Tuj1, pAKT-S473 and their merged images, and phosphorylation of S6 and GSK3β-S9 in a separate retina sample. In our experience, only overexpression of AKT can achieve this reliable pGSK3β S9 labeling. Scale bar, 20 µm. **B,** Mean fluorescence intensities of pAKT-S473 and **C,** Mean fluorescence intensities of pGSK3β-S9, Data are presented as means ± s.e.m, n=3. *: p<0.05, **: p<0.01 versus Pten KO + AKT3. One-way ANOVA with Tukey’s multiple comparisons post hoc test.
